# Exogenous short-term silicon application regulates macro-nutrients, endogenous phytohormones, and protein expression in *Oryza sativa* L.

**DOI:** 10.1186/s12870-017-1216-y

**Published:** 2018-01-04

**Authors:** Soo-Won Jang, Yoonha Kim, Abdul Latif Khan, Chae-In Na, In-Jung Lee

**Affiliations:** 1 Natural Resources Research Institute, R&D Headquarters, Korea Ginseng Corporation, Daejeon, 34128 South Korea; 2 Division of Plant Biosciences, Kyungpook National University, Daegu, 41566 South Korea; 3 UoN Chair of Oman’s Medicinal Plants & Marine Natural Products, University of Nizwa, 616 Nizwa, Oman; 4 Department of Agronomy, Gyeongsang National University, Jinju, 52828 South Korea; 5 Crop Physiology Laboratory, Division of Plant Biosciences, Kyungpook National University, Daegu, 41566 South Korea

**Keywords:** Silicon application, Gibberellins, Jasmonic acid, Glucose-6-phosphate isomerase, Importin alpha 1b, Protein expression, Radioisotope ^45^Ca

## Abstract

**Background:**

Silicon (Si) has been known to regulate plant growth; however, the underlying mechanisms of short-term exogenous Si application on the regulation of calcium (Ca) and nitrogen (N), endogenous phytohormones, and expression of essential proteins have been little understood.

**Results:**

Exogenous Si application significantly increased Si content as compared to the control. Among Si treatments, 1.0 mM Si application showed increased phosphorus content as compared to other Si treatments (0.5, 2.0, and 4.0 mM). However, Ca accumulation was significantly reduced (1.8- to 2.0-fold) at the third-leaf stage in the control, whereas all Si treatments exhibited a dose-dependent increase in Ca as determined by radioisotope ^45^Ca analysis. Similarly, the radioisotope ^15^N for nitrogen localization and uptake showed a varying but reduced response (ranging from 1.03–10.8%) to different Si concentrations as compared to ^15^N application alone. Physiologically active endogenous gibberellin (GA_1_) was also significantly higher with exogenous Si (1.0 mM) as compared to GA_20_ and the control plants. A similar response was noted for endogenous jasmonic and salicylic acid synthesis in rice plants with Si application. Proteomic analysis revealed the activation of several essential proteins, such as Fe-S precursor protein, putative thioredoxin, Ser/Thr phosphatase, glucose-6-phosphate isomerase (*G6P*), and importin alpha-1b (*Imp3*), with Si application. Among the most-expressed proteins, confirmatory gene expression analysis for *G6P* and *Imp3* showed a similar response to those of the Si treatments.

**Conclusions:**

In conclusion, the current results suggest that short-term exogenous Si can significantly regulate rice plant physiology by influencing Ca, N, endogenous phytohormones, and proteins, and that 1.0 mM Si application is more beneficial to plants than higher concentrations.

**Electronic supplementary material:**

The online version of this article (10.1186/s12870-017-1216-y) contains supplementary material, which is available to authorized users.

## Background

During the past several decades, scientists have focused on the role of silicon (Si) in various crops, such as barley [1, 2], cucumber [3], maize [1], rice [4–7], soybean [8], and tomato [9]. Si is the second most abundant element in soil, following oxygen [10], and is found as silica (SiO_2_), silicic acid (H_4_SiO_4_), and silicate (xM^1^_2_OySiO_2_) because of its strong affinity for other ions [11]. Higher plants usually take up aqueous silicic acid from the rhizosphere through their roots [12]. In rice plants, Ma et al. [7] reported that two specific genes (*OsLsi1* and *OsLsi2*) were involved in Si transfer between the rhizosphere and xylem. Si enters the shoot part with the help of *OsLsi6*, transporting Si into the xylem parenchyma region and then into the silicified cells of leaf blades [13]. During transport of Si, transport-related genes are activated and calcium (in Ca^2+^ form) is actively taken up through the solute gradient in either an apoplastic or a symplastic manner [14, 15]. In most cases, both Si and Ca are transported via the Casparian strip [15]. Cytosolic Ca^2+^ contributes to various physiological responses, such as opening stomatal guard cells, phototropism, and responses to gravity [16]. In addition, nitrogen (N) uptake, transportation, and distribution influence plant cell physiology and growth [17, 18]. Some recent studies have demonstrated ameliorative actions of N and Si [19–21]. The endogenous and exogenous Si and Ca^2+^ oscillation perturbs plant growth dynamics and responses to stress conditions [22].

In plants, Si absorbed from the soil creates silica cells or silica bodies and has been shown to confer tolerance to various biotic and abiotic stresses [13]. In addition to the role of Si in enhancing resistance to environmental stress, it can induce various physiological responses, such as regulation of antioxidant activity, modulation of endogenous hormones, alteration of mineral uptake, and promotion of adventitious shoot regeneration [11, 23–25]. Particularly under field conditions, Si application to rice not only induces an increase in plant height and culm length by upregulation of bioactive gibberellin (GA), but also reinforces cell walls. Subsequently, rice plants have greater photosynthetic efficiency and these physiological responses directly affect yield components [10, 23, 26–28].

The role of Si in higher plants has been well-described in various studies [4–7, 10, 29]. In particular, research related to Si-mediated abiotic and biotic stress amelioration is substantial [10]. For example, cadmium uptake was significantly reduced in Si-treated rice plants because of the regulation of P1-type ATPase [4]. Si application to wheat seedlings also resulted in UV stress mitigation through regulation of antioxidant activities such as ascorbate peroxidase (APX), catalase (CAT), and superoxide dismutase (SOD) [30]. In addition to its effects related to abiotic stress, Si can induce resistance against powdery mildew diseases in cucumber, barley, and strawberries, as well as suppress the spread of rice blast disease [31]. Previous studies have shown that Si can mitigate diverse abiotic and biotic stress; however, the underlying influences and changes in mineral uptake, especially Ca and N localization and uptake, have been little understood. Additionally, the effects of short-term application of different Si concentrations on the expression of specific proteins and genes are poorly described. The aim of this study was to elucidate the effects of short-term exogenous Si application on the uptake of Ca and N as well as on the regulation of endogenous phytohormones (gibberellins [GA], jasmonic acid [JA], and salicylic acid [SA]) and related protein expression in rice plants.

## Methods

### Plant materials and growth conditions

*Oryza sativa* L. ‘Dongjin’ seeds were procured from the National Institute of Crop Science, Rural Development Administration, The Republic of Korea, to perform Si-related experiments. The seeds were sterilized with 5% sodium hypochlorite for 10 min and thoroughly washed with autoclaved double-distilled water (DDW). After 3 days of soaking in DDW, germinated rice seedlings were transplanted into thoroughly autoclaved sand medium and placed into a growth chamber (KGC-175 VH, Koencon, South Korea) for 2 weeks to obtain uniform seedling growth. During the experiment, growth chamber conditions were adjusted to 12-h light (08:00–20:00 h, 30 °C, relative humidity 70%) and 12-h dark (20:00–08:00 h, 25 °C, relative humidity 70%). Yoshida solution was used as the nutrient source [32]. The solution pH was maintained daily at 5.0–5.3 by adding HCl to the medium to reduce the polymerization of silicates [5]. After 2 weeks, fully grown rice plants were transplanted to pots (25 cm × 20 cm × 20 cm) and supplied with DDW for 3 days to remove nutrients and sand medium. The rice plants were then used as experimental material.

### Silicon application to rice plants

The rice plants were treated with four different concentration of Si (Na_2_SiO_3_.9H_2_O; 0.5 mM, 1.0 mM, 2.0 mM, and 4.0 mM) in 4 L DDW for 24 h in the growth chamber as described earlier. The rice plant samples were harvested at three time periods: 6 h, 12 h, and 24 h following Si application. Each treatment had three replications and each replicate comprised 24 plants. All the samples were harvested in liquid nitrogen to perform phytohormonal, proteomics, and biochemical analysis. Each analysis was replicated three times.

### Determination of mineral content after Si application

The rice plants (1.0 g) were soaked in 0.5 M HCl for 20 s, rinsed with DDW, and dried for 72 h in an oven at 70 °C. Samples were weighed, ground to fine powder, and digested in 5 mL of a tertiary mixture of HNO_3_:H_2_SO_4_:HClO_4_ (10:1:4, *v*/v). The Si content was determined by inductively coupled plasma mass spectrometry (Optima 7900DV, Perkin-Elmer, Waltham, MA, USA) as shown by [5]. The experiment was repeated three times.

### Determination of endogenous phytohormones

The levels of GA and JA from whole shoot samples were determined. For GA analysis, we used 0.5 g freeze-dried plant samples. The extraction and quantification of GA_20_ and GA_1_ were conducted using an established protocol [33]. Deuterated GA_20_ (^2^H_2_ GA_20_) and GA_1_ (^2^H_2_ GA_1_) were used as internal standards, and as such, 20 ng of each standard was added during the extraction process. A 0.5 g of plant sample was extracted with 80% and 100% methanol (MeOH). This solution was refrigerated for 1 h at −70 °C and then filtered by a GF/A filter to remove chlorophyll. The extracted sample was passed through a 5 g C18 column (90–130 μm; Alltech, Deerfield, IL, USA) and dried on 1 g Celite. The dried Celite was loaded into 5 g of silicon dioxide (SiO_2_). Additionally, the extracted sample was mixed with polyvinylpolypyrrolidone (PVPP, Sigma Aldrich, USA) for 1 h and then the mixture was filtered. The extracted sample was partitioned three times with equal volumes of EtOAc. The EtOAc fraction was dried in a vacuum, and the residue was dissolved in 4 mL of 100% MeOH. This solution was dried using nitrogen gas [34]. The extracted residue was quantified by a GC-MS-SIM (Hewlett-Packard 6890, 5973 N mass selective detector, Agilent, USA) equipped with a HA-1 capillary column (30 m × 0.25 mm i.d. 0.25 μm film thickness). Details of the GC-MS-SIM operation protocol are given in Additional file 1: Table S1. The endogenous GA_20_, and GA_1_ contents were calculated from the peak area ratios of 418/420 and 506/508, respectively (Additional file 2: Figure S1), wherein the data are provided in nanograms per gram dry weight and the data were collected from three replications.

To extract endogenous JA, we used 0.5 g freeze-dried plant samples. The JA was extracted according to the protocol of McCloud and Baldwin [35]. A total of 0.5 g dried sample was extracted with a solution of acetone and 50 mM citric acid (70:30 *v*/v), and [9, 10-^2^H_2_] 9, 10-dihydro-JA (20 ng) was added as an internal standard. The aqueous solution was then filtered and extracted with 10 mL of diethyl ether three times and loaded into a solid phase extraction cartridge (Sep-Pak Cartridge, Waters, USA). The cartridges were washed with 7.0 mL of trichloromethane and 2-propanol (2:1 v/v). Both JAs (endogenous JA and the internal standard) were eluted with 10 mL of diethyl ether and acetic acid (98:2 v/v). The sample was adjusted to 50 μL with dichloromethane after evaporation of solvents and etherification of the residue with excess diazomethane. Then, the extracts were analyzed by GC-MS-SIM using the same equipment as in the GA analysis. We monitored ion fragments (*m/z* 83) corresponding to the base peaks of JA and [9, 10-^2^H_2_]-9, 10-dihydro-JA for JA determination (Additional file 3: Figure S2). On the basis of the peak area of both JAs (standard and endogenous JA), we calculated JA content with the following formula: JA = (endogenous JA) / (standard JA) × standard amount / sample weight. All data were represented by three replications.

Endogenous free SA was measured by high-performance liquid chromatography (HPLC), and the conditions are described in Additional file 1: Table S1. Free SA was extracted based on Enyedi et al. [36] and Seskar et al. [37]. A 0.2 g freeze-dried sample was sequentially extracted with 90% and 100% methanol in a centrifuge (10,000×*g*). Both extracts were dried in a vacuum. The dry pellets were resuspended in 2.5 mL of 5% trichloroacetic acid and the supernatant was partitioned with ethyl acetate/cyclopentane/isopropanol (49.5:49.5:1, *v*/v). The top layer was transferred to a 4 mL vial and dried with purified N gas. The SA was again suspended in 1 mL of 70% methanol and analyzed by HPLC.

### Bio-image of calcium distribution

To analyze calcium distribution in rice leaves, we applied different concentrations of Si with 400 Bq of radioisotope ^45^Ca (^45^CaCl_2_, 37 MBq, NEN, USA). Thus, our experiment was composed of five different treatments, including i) DDW alone, ii) 0.5 mM Si + ^45^Ca, iii) 1.0 mM Si + ^45^Ca, iv) 2.0 mM Si + ^45^Ca, and v) 4.0 mM Si + ^45^Ca. Calcium (Ca) distribution was analyzed using a bio-imaging analyzer. We removed roots from Si and ^45^Ca-treated rice seedlings after 48 h, and then plant samples were dried at 85 °C for 12 h. Dried plant samples were sealed with high-density thin film (Singsing Wrap™, LG Co., South Korea) to prevent sample destruction or contamination. For 24 h, plant samples were placed on cassettes (BAS-MS 2040, Fuji, Japan) containing an imaging plate (IP) that was covered with polyester. After this, reactive IP was analyzed by an IP reader (BAS-MS 1500, Fuji, Japan) and the collected data were read by an image analysis program (TINA). Finally, data were illustrated with different colors according to the concentration of ^45^Ca [38].

### Determination of ^45^Ca and urea-^15^ N

The dry weight of the plant samples was measured and samples were ground in a crucible for Ca isotope analysis. The crucibles were placed in an electronic furnace at 500 °C for 12 h. Plant ash samples were mixed with a cocktail solution (Ready Organic, Beckman, USA) for a liquid scintillation counter (LSC), and radiation activity was measured by the LSC (LC 1801, Beckman, USA). To analyze nitrogen uptake, we applied urea-^15^ N (2.0 mM, 4.0 mM, and 8.0 mM) and urea-^15^ N with 1.0 mM Na_2_SiO_3._ Next, samples were harvested at 6 h and 12 h after exposure. Untreated rice plants (with only the application of distilled water) were used as control plants. Shoot samples were dried at 75 °C for analysis. Dried-shoot samples were ground into powder and passed through a 150 μm sieve to analyze isotope ratios in a mass spectrometer (Delta V Advantage, Thermo Fisher Scientific Inc., USA). The experiment was repeated three times.

### Protein extraction, 2DE, and MALDI-TOF MS analysis

Protein was extracted from the culture medium. We added a five-fold volume of acetone to the culture medium. For protein precipitation, the mixture of acetone and culture medium was refrigerated at −20 °C for 2 h. The precipitated proteins were collected by centrifuge and collected proteins were eluted by a solution containing 7 M urea, 2 M thiourea, 4% (*w*/*v*) 3-[(3-cholamidopropyl) dimethyammonio]-1-propanesulfonate (CHAPS), 1% (w/v) dithiothreitol (DTT), 2%(*v*/v) pharmalyte, and 1.0 mM benzamidine. Concentrations of protein were attained following the method of Bradford [39].

The samples were diluted with re-swelling solution (7 M urea, 2 M thiourea, 2% 3-[(3-cholamidopropy) dimethyammonio]-1-propanesulfonate (CHAPS), 1% dithiothreitol (DTT), and 1% pharmalyte) at room temperature for 12–16 h. Isoelectric focusing (IEF) was conducted according to the manufacturer’s manual (IPGphor II system, Amersham Biosciences, USA) and the conditions were 207 °C with a current limit of 50 mA/strip: 3 h at 300 V, 6 h at 1000 V, 3 h at 8000 V (gradient), and 24 h at 8000 V. To conduct the second dimension (SDS) analysis, the individual strips were equilibrated for 15 min in 10 mL equilibration solution (6 M urea, 30% glycerol, 2% SDS, 0.002% bromophenol blue, and 50 mM Tris pH 8.8) containing 1% *w*/*v* DTT and subsequently for 15 min in 10 mL equilibration buffer containing 4.5% w/v iodoacetamide.

Proteins were visualized by silver staining [40]. The gels were fixed in fixing solution (40% ethanol and 10% acetic acid) overnight for silver staining. The gels were consecutively washed with 30% ethanol for 20 min, 20% ethanol for 20 min, and DDW for 20 min, and then were consecutively sensitized, developed, and neutralized. Detailed information is described in Carpentier et al. [41]. Stained gels were scanned using the Duoscan T1200 (Agfa, USA) and images were analyzed by PDQuest software (version 7.0, BioRad) for quantification. Individual spot quantity was normalized by total valid spots, and spot detection was realized without spot editing. The spots were quantified using the % volume criterion, and significantly different spots (two-fold difference) were used for identification.

Each protein was analyzed by Ettan MALDI-TOF (Amersham Biosciences, UK) for identification. Protein fragments were evaporated at 337 nm using an N_2_ laser and were accelerated by a 20 kV injection pulse. The mass spectrum of each spot was measured by accumulated peaks, which were collected by 300 nm laser shots. The ion peak m/z (842.510, 2211.4016) was used as the standard peak for mass spectrum, and we used Profound (http://prowl.rockefeller.edu/), which was developed by The Rockefeller University, for protein identification.

### RNA extraction and RT-PCR analysis

The total RNA was extracted from plant samples using an RNeasy Plant Extraction Mini Kit (Qiagen) following the manufacturer’s instructions. For Reverse-Transcription (RT)-PCR, complementary DNA was synthesized using an RT Kit (Solsent, Korea). All the reactions were performed according to the manufacturer’s instructions. RT-PCR was conducted for evaluation of mRNA expression of *G-6-P* and *IMP3* [42]. *OsUBI* was used as a control. The primers for RT-PCR are mentioned in Additional file 4: Table S2.

### Statistical analysis

To evaluate quantitative differences in radioisotope ^45^Ca content and plant hormones, all data were statistically analyzed for standard errors using Sigma Plot Software (2004). A two-way analysis of variance (ANOVA) was performed to determine significant differences among results (SAS release 9.1; SAS, Gary, NC, USA), and differences between mean values among treatments were compared using Duncan’s multiple range test (DMRT) at *P* < 0.05 and *P <* 0.01. All the analyses were performed in triplicate.

## Results

### Influence of mineral uptake after Si treatment

To evaluate mineral uptake in rice plants, we measured several mineral ratios after Si application (0.5, 1.0, 2.0, and 4.0 mM) in hydroponically grown rice plants. The nitrogen (N) and potassium (K) ratio showed a decreasing pattern as compared to the control, however, it was not statistically different (*P* < 0.05) (Table 1). The phosphorus (P) ratio in Si-applied plants revealed a slight decrease in the pattern, except in the 1.0 mM Si treatment. Conversely, the magnesium (Mg) ratio was significantly increased in the 1.0, 2.0, and 4.0 mM Si treatments in comparison to the control, but no difference was observed in the 0.5 mM Si treatment as compared to the control (Table 1). The calcium (Ca) ratio decreased approximately to 11.8% (0.5 mM and 4.0 mM) from 15.8% (1.0 mM) in all concentrations of Si treatments. The Si content was absolutely increased in a dose-dependent manner when Si was applied to plants. This supports the idea that changes in mineral contents were triggered by the Si applications (Table 1).

**Table 1:** Effect of exogenously applied Si on mineral content in rice plants. Si was applied in hydroponic solution (Yoshida solution) for 24 h and then samples were collected for analysis

Treatments	T-N (%)	P (%)	K (%)	Ca (%)	Mg (%)	Si (ppm)
Control	1.33±0.10a	0.24±0.01a	1.95±0.28a	0.19±0.11a	0.08±0.01b	3007±12.3b
0.5 mM Si	1.34±0.12a	0.22±0.09ab	1.93±0.31a	0.17±0.21ab	0.09±0.01b	3155±8.91ab
1.0 mM Si	1.28±0.27a	0.25±0.1a	1.92±0.13a	0.16±0.15ab	0.11±0.03ab	3402±13.4a
2.0 mM Si	1.27±0.0.21a	0.21±0.1b	1.92±0.42a	0.15±0.02b	0.10±0.08ab	3440±21.2a
4.0 mM Si	1.27±0.13a	0.22±0.1ab	1.90±0.23a	0.17±0.06ab	0.12±0.02a	3431±11.49a

Values with ± shows the standard error of the mean of three replications. Different letters in each column indicate statistically significant differences (*P* <0.05) among treatments by Duncan's Multiple Range Test (DMRT)

### Influence on endogenous hormone levels after Si treatment

**Fig. 1:**
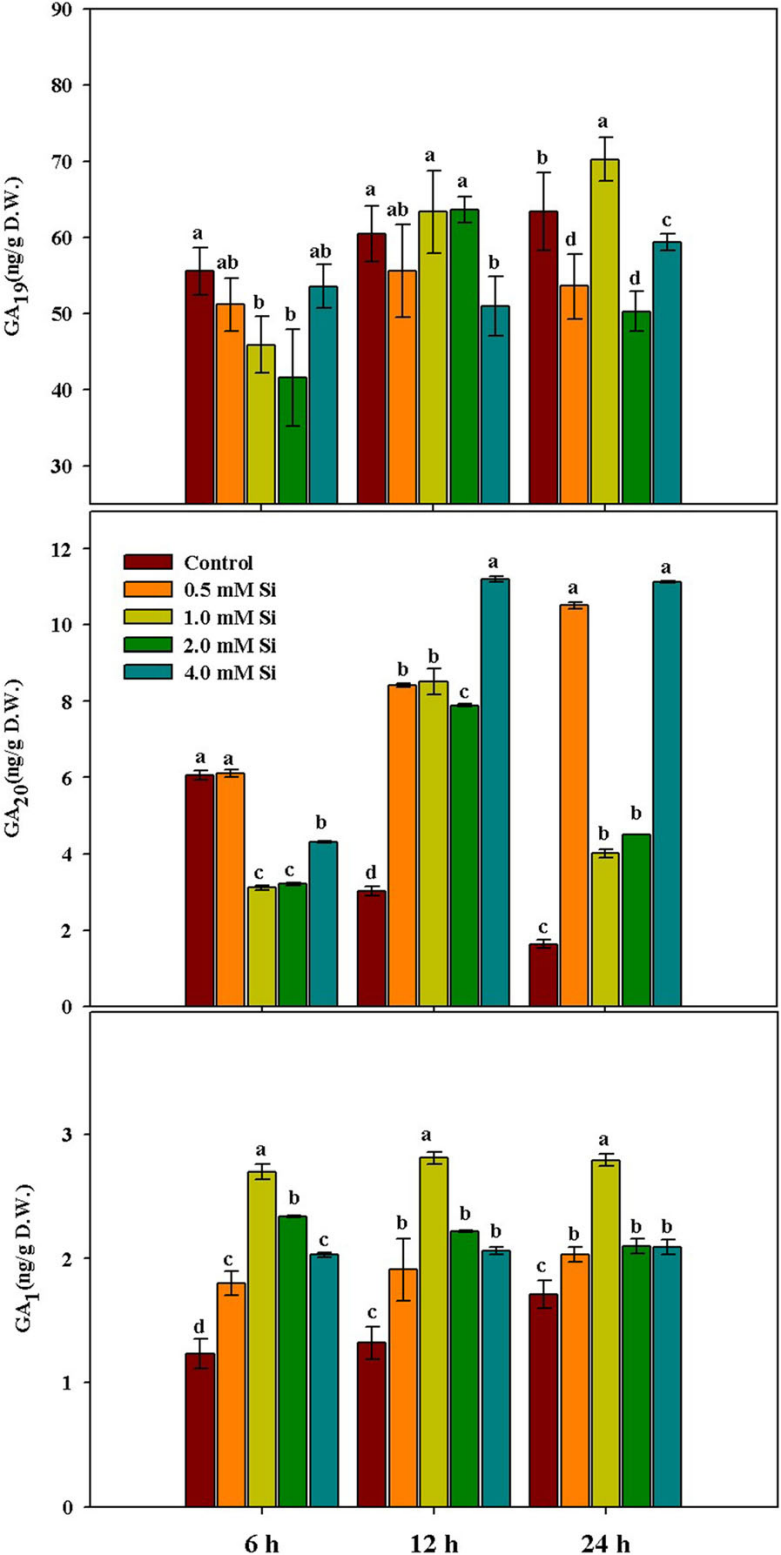
Changes in the levels of gibberellin (GA_1_) and its precursors (GA_19_ and GA_20_) in rice plants in response to different concentrations of Si. The different letter(s) indicates significant differences at *P* < 0.05 according to Duncan’s multiple range test (DMRT). Each bar shows the standard error of the means of three replications

**Fig. 2:**
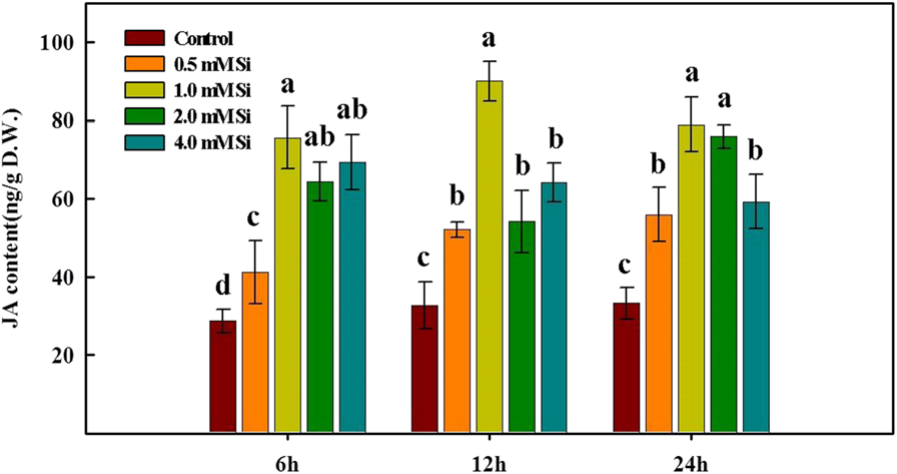
Regulation of endogenous jasmonic acid (JA) levels in rice plants after the exogenous application of different concentrations of Si. The different letter(s) indicate significant differences at *P* < 0.05 according to Duncan’s multiple range test (DMRT). Each bar shows the standard error of the means of three replications

**Fig. 3:**
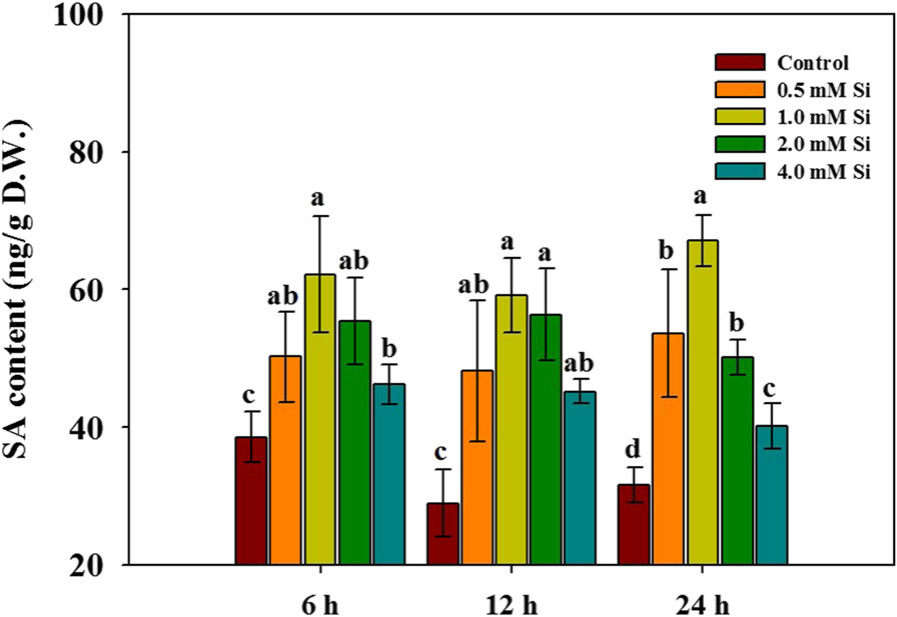
Influence of different concentrations of Si application on endogenous salicylic acid (SA) levels in rice plants. In the figure, different letters indicated significant differences at *P* < 0.05 according to Duncan’s multiple range test (DMRT). Each bar shows the standard error of the means of three replications

Plant hormones are known to induce various physiological and biochemical responses at very low concentrations [33]. For this reason, we analyzed endogenous GA and JA levels after Si application. Bioactive GA_1_ concentration was significantly increased with Si application (0.5, 1.0, 2.0, and 4.0 mM) for all exposure times (6 h, 12 h, and 24 h) as compared to that of the control (Fig. 1). In particular, comparison of GA_1_ levels among Si treatments revealed that it was significantly higher (*P* < 0.01) in the 1.0 mM Si treatment as compared to that of the remaining Si treatments (Fig. 1). Concentrations of GA_20_ also exhibited higher levels with Si application as compared to the control at 12 h and 24 h exposure, whereas GA_20_ content was significantly decreased following Si application, except for the 0.5 mM Si treatment, in comparison with that of the control at 6 h exposure (Fig. 1). Endogenous hormone JA also exhibited a similar pattern as GA_1_. Endogenous JA levels were significantly increased at all concentrations of Si application as compared to that of the control, and this tendency was consistently observed in all periods (Fig. 2). Comparison of JA content among Si treatments revealed a maximum value with 1.0 mM Si treatment, whereas 0.5 mM Si induced the minimum value in all time periods. There were no differences in response between 2.0 mM Si and 4.0 mM Si treatments at 6 and 12 h exposure (Fig. 2). Similarly, increased hormone patterns following Si application were detected. The 1.0 mM Si treatment exhibited highly increased endogenous free SA content as compared to that of the control and other concentrations of Si treatments (Fig. 3). Comparison of free SA content among different Si treatments revealed that the maximum content was observed with 1.0 mM Si application, while 0.5 mM Si and 2.0 mM Si resulted in similar SA levels (Fig. 3). Low free SA levels were detected following the 4.0 mM Si treatment for all periods, although it remained at a higher level than that of the control (Fig. 3).

### Distribution and concentration of ca in rice leaves

**Fig. 4:**
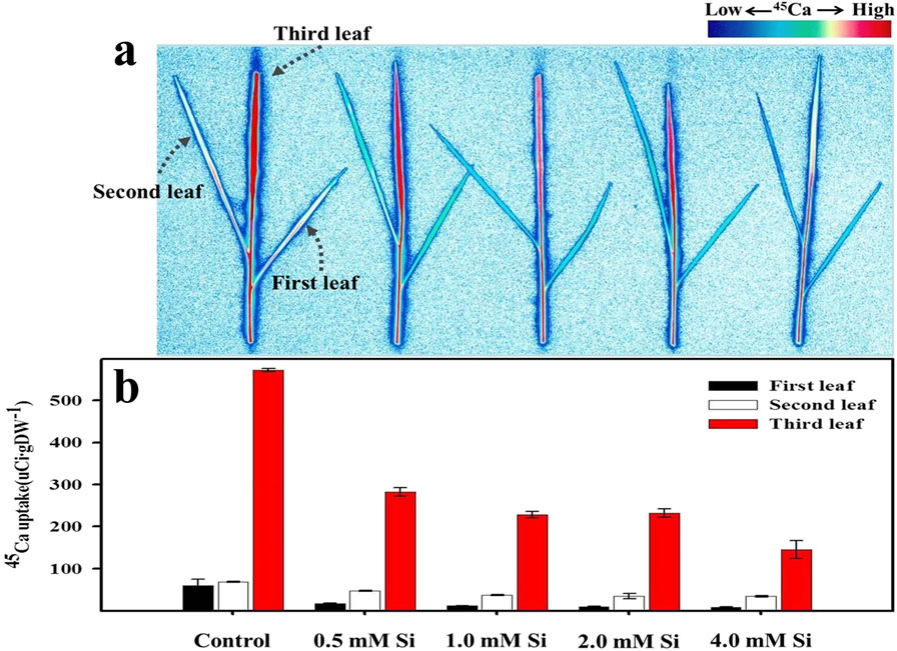
Changes in calcium uptake under Si application in rice plants. In Figure (**a**), the color closest to red indicates higher uptake of ^45^Ca, whereas the color closest to blue indicates lower uptake of ^45^Ca. Figure (**b**) shows quantities of ^45^Ca at different leaf positions. Each bar shows the standard error of the means of three replications

In our previous experiment, we found that Ca uptake in rice plants decreased with increasing concentrations of Si. To identify Ca movement and uptake, we directly applied radioisotope ^45^Ca during the application of varying Si concentrations (0.5, 1.0, 2.0, and 4.0 mM) to hydroponically grown rice plants. The radioisotope data are shown in Fig. 4. According to the image for ^45^Ca accumulation and localization, the third leaf showed high ^45^Ca accumulation as compared to the first and second leaf, and this pattern was consistent in the control and all Si treatments (Fig. 4a). In case of the third leaf, however, ^45^Ca accumulation was highly reduced in Si-treated plants as compared to the control. Additionally, ^45^Ca accumulation in the third leaf was gradually reduced with increasing Si concentration (Fig. 4a). The content of radioisotope ^45^Ca was significantly inhibited (approximately 1.8- to 2.0-fold reduction in comparison to the control) by all exogenous Si applications (Fig. 4b).

### Effects of Si on N uptake and distribution in rice leaves

**Fig. 5:**
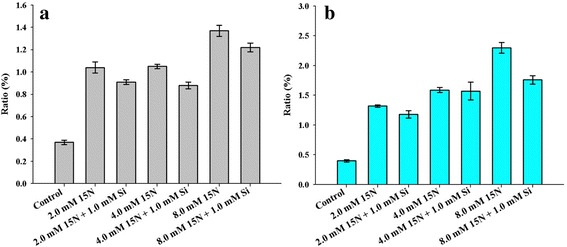
Effects of Si application on nitrogen uptake in rice plants. Figure (**a**) shows the ^15^N ratio in rice plants at 6 h exposure and Figure (**b**) indicates the ^15^N ratio in rice plants at 12 h exposure. Each bar shows the standard error of the means of three replications

We applied different concentrations of urea-^15^ N (2.0 mM, 4.0 mM, and 8.0 mM) and urea-^15^ N with 1.0 mM Si for 12 h and the samples were harvested twice (6 h and 12 h). The ^15^N ratios are shown in Fig. 5. In the 6 h treatment, the ^15^N ratio was increased in all treatments in comparison with the control. When we applied ^15^N (2.0, 4.0, and 8.0 mM) with 1.0 mM Si, the ^15^N ratio was reduced as compared to ^15^N treatment alone (Fig. 5a). In the case of the 12 h treatment, the same trend and effect were observed. The ^15^N ratio was increased in ^15^N–supplemented rice plants as compared to the control, whereas the ^15^N ratio was slightly decreased in the ^15^N with Si-treated rice plants in comparison to plants treated with ^15^N alone (Fig. 5b).

### Influence of Si application with nitrogen and calcium fertilizer on mineral uptake

Our previous results showed that N and Ca uptake were significantly decreased by Si application. However, the previous study was conducted under restricted environmental conditions (only ^15^N and ^45^Ca supplied). Thus, we conducted an analysis of mineral uptake in rice plants after a combination of nitrogen (NH_4_NO_3_) and calcium fertilizer (CaCl_2_) treatments with Si (Additional file 5: Table S3). Total nitrogen (T-N) content was significantly increased in the NH_4_NO_3_ treatment in comparison to that of the control; however, it was suppressed by approximately 10.6% by the addition of 1.0 mM Si (Additional file 5: Table S3). Conversely, T-N content did not exhibit a statistically significant difference among independent and combination treatments of CaCl_2_ and Si (Additional file 5: Table S3). With NH_4_NO_3_ treatment, other mineral contents, such as those of P, K, Ca, and Mg, did not exhibit any difference as compared to the control, whereas Si concentration significantly increased in NH_4_NO_3_ with Si application, as expected (Additional file 5: Table S3). In the CaCl_2_ and Si combination treatment, Ca uptake was significantly inhibited by 1.0 mM Si supplement in comparison with that of CaCl_2_ alone (Additional file 5: Table S3). Except for Si concentrations, we did not observe statistically increased or decreased mineral uptake among any of the treatments (Additional file 5: Table S3).

### Influence of Si on protein expression

**Fig. 6:**
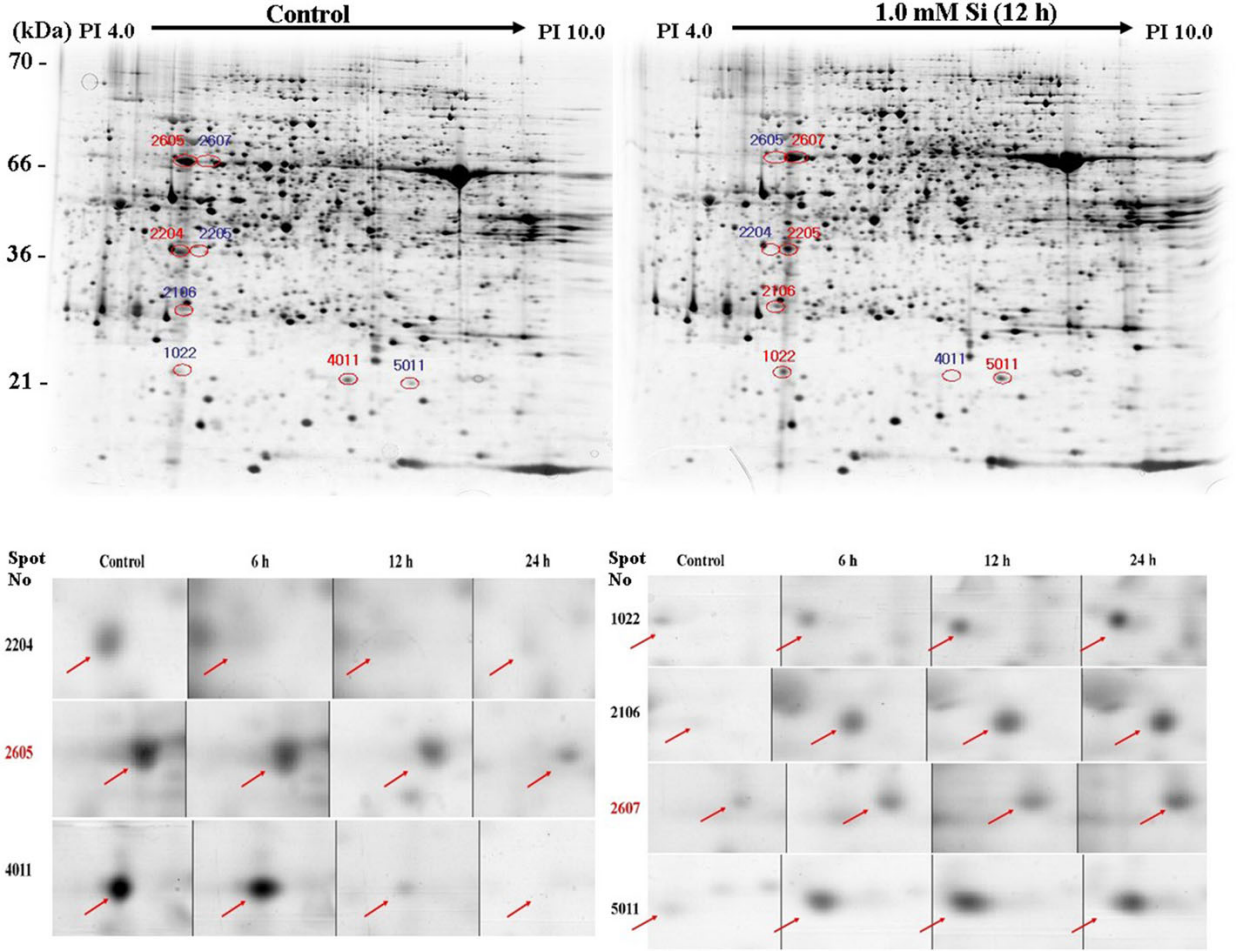
Expressions of protein levels in rice plants after exposure to short-term Si application. In the figure, red circles reveal upregulated protein spots as compared to that of the control, and blue circles indicate downregulated protein spots relative to that of the control

Si application to rice plants inhibited N and Ca uptake as well as increased endogenous hormones. Thus, we conducted 2-DE gel analysis to determine the difference in protein expression between non-Si treatment and 1.0 mM Si treatment. Different protein expressions are shown in Fig. 6. According to our results, seven kinds of 2-DE spots exhibited significant (*P* < 0.01) differences between Si-treated and Si-untreated plants (Fig. 6). Among the seven protein spots, four were upregulated in Si-treated plants (Spot No: 1022, 2106, 2607, and 5011), and the other three were downregulated (Spot No: 2204, 2605, and 4011) (Table 2). All spots’ (1022, 2106, 2204, 2605, 2607, 4011, and 5011) information is very well described in Table 2. Among these seven spots, spot 2607 was significantly upregulated by 1.0 mM Si treatment, and it was identified as importin alpha 1b protein. Conversely, spot 2605, glucose-6-phosphate isomerase, was significantly downregulated by 1.0 mM Si treatment and was significantly higher across treatments (Table 2).

**Table 2:** Different protein expression between Si treated and Si untreated plant. Protein information was identified by mass and plant samples harvested at 12 h after Si application

Spot No	Protein name	Exp MW/PI	Theo MW/PI	SC (%)	Expression Pattern	Accession No
1022	Rieske Fe-S precursor protein	21.09/4.93	24.21/9.2	22	up	gi|50,508,582
2106	Putative thioredoxin-like protein CDSP32	29.26/5.01	32.48/6.3	34	up	gi|34,901,636
2204	Putative Ser/The protein Phosphatase	36.54/5.00	35.26/5.1	19	down	gi|34,911,376
2605	Putative glucose-6-phosphate isomerase	64.17/5.01	68.86/5.7	20	down	gi|50,900,276
2607	Importin alpha 1b	62.30/5.03	59.09/5.2	26	up	gi|6,682,927
4011	unnamed protein product	21.08/5.56	15.90/5.7	25	down	gi|34,895,594
5011	Rieske Fe-S precursor protein	20.89/6.20	24.21/9.2	17	up	gi|50,508,582

Expression pattern indicated a comparison with control. Accession number (GI number), *MW* Molecular weight, *PI* Isoeletronic point, *SC* Sequence coverage, *Theo* Theoretical value

### Influence of Si on expression of importin and the G-6-P gene

**Fig. 7:**
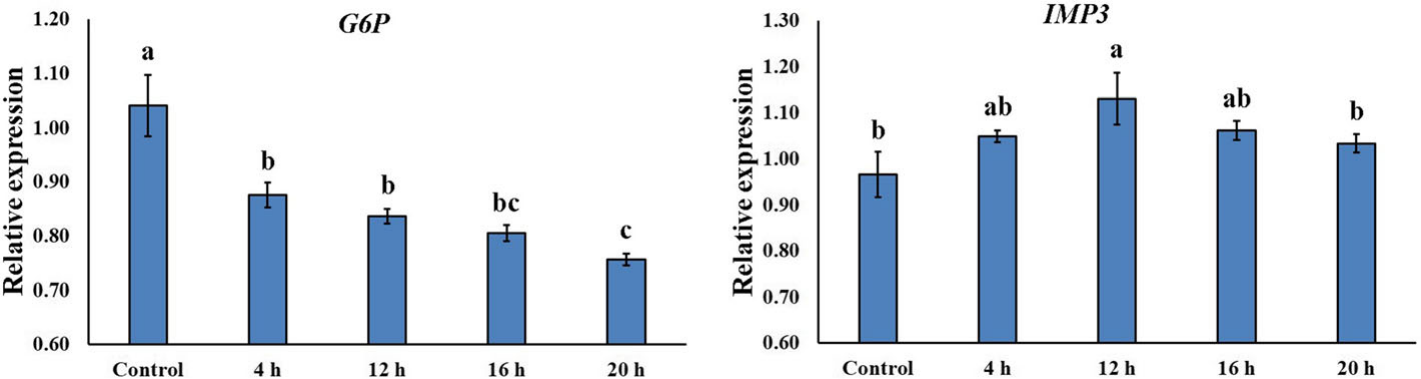
The mRNA expression of *G6P* and *IMP3* in rice plants after Si application. Plant samples were collected at 4 h, 12 h, 16 h and 20 h after Si treatment. In the figure, a vertical bar means standard ± standard deviation (*n* = 3)

We found different protein expressions after Si application. Thus, we compared the mRNA expression of *IMP3* and *G-6-P* genes in the control and 1.0 mM Si-treated plants by RT-PCR analysis. The mRNA expression of *IMP3* was increased in Si-treated rice plants as compared to that of the control (Fig. 7). Conversely, mRNA expression of *G-6-P* exhibited a decreasing pattern in comparison with that of the control; in particular, mRNA expression level gradually decreased with increasing Si exposure period (Fig. 7).

## Discussion

Exogenous Si application has been reported to confer ameliorative potential to the growth and physiology of a wide array of crop plants. Several studies have shown beneficial Si effects such as intervention in oxidative stress, phytohormonal changes, gene expression, and resistance against abiotic and biotic stresses [10, 11, 25, 30, 31, 43, 44]. Recently, Deshmukh et al. [45] reported the existence of a Si uptake gene in dicotyledonous plants. The gene was located in aquaporin, and thus it reached the plant cell by passing through aquaporin in water [45]. Therefore, uptake of Si can induce various physiological responses. Our first experiments on Si focused on the effects of Si on plant growth and crop yield. According to Datnoff et al. [46], Japanese farmers increased rice productivity (6 t/ha) by integrating nutrient management, including Si fertilization.

Essential elements, such as N, P, K, Mg, and Ca, are known to be key regulators of plant life. Nitrogen (N) is an essential macronutrient and is a component of amino acid, amides, proteins, nucleic acids, and nucleotides, as well as a constituent of nitric oxide (NO), which is a highly reactive free radical that is involved in the immune system in animal and plant cells [47–49]. Therefore, under serious N deficiencies, plants exhibit chlorosis (yellowing of the leaves) owing to their inability to produce amino acids and other metabolites that are essential for metabolic processes [50–52]. In addition, N is involved in physiological responses, such as the growth of stem internodes, leaf expansion, and acceleration of cell division [23]. In particular, rice plants quickly respond to exogenous N sources in soil. Thus, rice plants can induce shoot growth. Si is absorbed by low-silicon genes (*OsLsi1, OsLsi2, HvLsi1,* and *HvLsi2*), which are located on both sides of the Casparian strip in rice and barley roots, and Si can be transferred to the shoots through the xylem [2].

N concentration did not exhibit a statistical difference with Si application alone (0.5 mM, 1.0 mM, 2.0 mM, and 4.0 mM) as compared to that of the control; however, the ratio of urea-^15^ N showed an inhibition pattern when N was applied with 1.0 mM Si. Thus, our results showed that Si application could inhibit N uptake in rice plants. However, our findings were different from those of previous studies. Cho et al. [53] analyzed nutrient recovery rates after they applied ^15^N and purified Si fertilizer to rice plants and found increased ^15^N uptake at higher concentrations of Si supplementation in rice plants. Jawahar and Vaiyapuri, [54] also reported that N uptake was increased when they applied more Si fertilizer to rice plants. However, both experiments mentioned above were conducted under field conditions. In addition, data were collected over long periods after Si fertilizer application to the rice (panicle initiation stage and harvesting stage) [53]. In contrast, we applied ^15^N with 1.0 mM Si simultaneously and then collected plant samples at 12 h and 24 h after treatment. This difference (different Si applications) resulted in different N uptake. Another possibility is that N uptake decreases with Si supplement at short exposure times (12 h and 24 h), but increases when rice plants are exposed to Si for long periods (30 days and 60 days). To acquire more evidence, additional experiments are needed in which purified Si is applied with and without ^15^N to rice plants and N uptake is traced for a long period.

According to our results, Ca uptake was significantly inhibited by Si application. This could be attributed to the fact that Si administered the stress cofactors, whereas Ca oscillation has mostly been credited to high-stress conditions [55]. The same results were observed by Ma and Takahashi [56]. They applied 100 ppm of SiO_2_ as silicic acid to hydroponically grown rice plants for 72 h and then harvested plant samples for nutrient analysis. They found that Ca uptake was significantly decreased (approximately 39%) by Si supplementation as compared to that of non-supplemented plants. However, different results were reported by other researchers [55, 57, 58]. According to Kaya et al. [57], Ca contents in leaves did not exhibit differences between control and Si-treated plants when Si was applied to maize grown under well-watered conditions, whereas increased Ca content was observed in Si-treated maize grown under water-limiting conditions as compared to non-Si-treated maize. In short, Ca uptake was increased with Si (K_2_SiO_3_) application to a relatively salt-tolerant cultivar under normal and salinity stress conditions [55, 58].

Plant hormones, such as GA and JA, are known to be signal molecules for various physiological responses at very low concentrations [11, 59]. GA is biosynthesized from trans-geranylgeranyl diphosphate in chloroplasts and then moved into the endoplasmic reticulum and cytosol. In particular, bioactive GA_1_ and GA_4_ are synthesized by two different pathways in the cytosol [60]. According to our results, bioactive GA_1_ concentration was significantly increased in Si-treated rice plants at all periods; however, GA_20_ content was increased at 12 h and 24 h after Si treatment. In particular, GA_20_ concentration exhibited a decreased pattern relative to that of the control. Focusing on the change in bioactive GA_1_, we showed only GA_20_ and GA_1_ data; however, we also analyzed the precursors of GA_20_ (GA_19_, GA_53_, and GA_12_). GA_19_ content exhibited an increase (50 ng to 60 ng, data not shown) relative to that of the control. Therefore, the difference between bioactive GA_1_ and non-active GA_20_ in control plants was probably induced by the activities of different types of enzymes. Similar results were also reported when ABA-treated oriental melons and their controls were compared. It was found that GA_20_ concentration gradually decreased, but GA_19_ concentration had an opposite and increasing trend in accumulation during the application period [61]. Normally, increased bioactive Gas (GA_1_ and GA_4_) contributes to stress mitigation (salinity stress: [8]; waterlogging: [62]), cell elongation during submergence [63] and root elongation [64]. Based on previous studies, therefore, we can hypothesize that Si application to rice can induce physiological or biochemical changes. Similar conclusions were also drawn by Kim et al. [11], where Si application significantly influenced endogenous plant hormones under either normal or stress conditions induced by salinity, heavy metals, and drought.

Another plant hormone, JA, can also trigger diverse physiological responses, such as floral development [65], senescence induction [66], potato tuberization [67], biotic stress amelioration [68], and growth inhibition [69]. In particular, JA plays an important role in plant defenses against insect herbivory and fungi [11]. Thus, JA can induce various defense proteins when plants are exposed to insect herbivory [29, 70]. When different concentrations of Si were applied to rice plants, endogenous JA content was significantly increased in all Si-treated plants as compared to that of non-Si treated plants. The way in which rice plants take up Si from soil to shoot has already been identified [7, 13]. Absorbed Si in rice plants can be immediately deposited in the leaf blade (2.5 μm) of rice and observed in a Si-cuticle double layer [71]. Therefore, we assumed that Si supplementation confers mechanical strength to rice plants and also induces a similar innate immunity against biotic stress factors, such as insects. Thus, endogenous JA contents were significantly increased in Si-applied rice plants. Similar results were reported by Kim et al. [72, 74]. They reported that JA levels were significantly increased in Si-applied rice plants under normal conditions (non-stress conditions); however, when they supplied mechanical wounding stress to rice plants, JA levels were strongly inhibited in Si-treated plants in comparison with that of the control. In addition, differences were observed at the genetic level that involved the JA biosynthesis pathway as compared to that of the control. In addition, JA enrichment was observed under stressful conditions [8, 11, 73, 74]; thus, our result assumed that exogenously applied Si could enhance resistance to biotic or abiotic stress via upregulated JA because high levels of JA could induce increased antioxidant (catalase: CAT) activity or decreased non-enzymatic antioxidant activity (malondialdehyde: MDA) [4, 25].

Si application can induce changes in phytohormones, such as GA_1_ and JA; thus, we evaluated Si effect on changes in proteins. Through our experiment, up- or down-regulated spots were identified. Among upregulated spots, spot 2607 was significantly upregulated by Si application and was identified as importin alpha 1b. Importin alpha protein binds to the nuclear localization signals (NLS) in the cytoplasm [75]. According to Jiang et al. [75], rice importin alpha 1 specifically binds to T-NLS and O2-NLS proteins, whereas Yamamoto and Deng [76] show binding to typical classes of NLS in *Arabidopsis* [77]. Thus, tissue-specific expression of importin could regulate protein importation into the nucleus and could participate in NLS-selective recognition [75]. Si-applied rice plants exhibited downregulated proteins; in particular, glucose-6-phosphate isomerase (*G6P*) was significantly downregulated. *G6P* and D-fructose 6-phosphate (*F6P*) are catalyzed from phosphoglucose isomerase, which is the second step in glycolysis as well as gluconeogenesis and the glycosylation of protein [78]. The effects on *G6P* and *F6P* showed that these are significantly downregulated by Si supplementation during glycolysis [79]. This is in agreement with the previous findings. In order to understand the underlying mechanism of Si application, further in-depth molecular studies are necessary to understand the key interactions.

## Conclusions

In summary, Si application to rice plants induced several physiological changes. First, Si-applied rice plants significantly inhibited Ca uptake. In particular, Ca uptake was significantly inhibited in relatively young leaves (third leaf) in comparison to older leaves (first leaf) and this phenomenon was confirmed by uptake of radioisotope ^45^Ca. Second, Si application could induce higher levels of bioactive GA_1_, JA, and SA. Thus, we assumed that endogenous hormone change would participate in various physiological responses, especially with increased hormones involved in the mitigation of abiotic stress. Third, Si treatment affected the expression of several proteins; in particular, importin alpha 1b protein was upregulated and *G6P* protein was downregulated by Si supplementation. Thus, we hypothesize that Si was able to regulate protein importation into the nucleus and also might be involved in regulation of *G6P* and *F6P* during glycolysis of rice plants. The current study will serve as the basis for further in-depth studies on the physio-chemo-proteomics of Si, while focusing on flooding and salinity stresses.

## Additional files

Additional file 1: Table S1. Conditions of GC-MS-SIM and HPLC for analyzing the plant hormones. (DOCX 14 kb)

Additional file 2: Figure S1. Information for GC-MS-SIM chromatogram. Bioactive GA_1_ showed two different ion values [^2^H_2_ GA_1_ (508 ion) was used as the standard and GA_1_ (506 ion) was used as endogenous GA_1_]. (DOCX 14 kb)

Additional file 3: Figure S2. Information for GC-MS-SIM chromatogram. Me-JA retention time was 18:46 and internal standard (9,10-^2^H_2_ JA) retention time was 18:70. (DOCX 15 kb)

Additional file 4: Table S2. The primers used for real-time PCR. (JPEG 107 kb)

Additional file 5: Table S3. Influence of mineral uptake in rice plants after lone nutrient treatment or each nutrient with Si application. All plant samples were exposed to the nutrient alone or in combination with Si for 24 h and then samples were analyzed for mineral uptake. (JPEG 107 kb)

## Abbreviations

APX: Ascorbate peroxidase
Ca: Calcium
CAT: Catalase
DDW: Double-distilled water
*F6P*: D-fructose 6-phosphate
*G6P*: Glucose-6-phosphate isomerase
GA: Gibberellins
*Imp3*: Importin alpha-1b
IP: Image plate
JA: Jasmonic acid
MDA: Malondialdehyde
Mg: Magnesium
N: Nitrogen
NLS: Nuclear localization signal
NO: Nitric oxide
SA: Salicylic acid
Si: Silicon
SOD: Superoxide dismutase
T-N: Total nitrogen

## References

[1] Mitani N, Chiba Y, Yamaji N, Ma JF (2009). Identification and characterization of maize and barley Lsi2-like silicon efflux transporters reveals a distinct silicon uptake system from that in rice. Plant Cell.

[2] Yamaji N, Chiba Y, Mitani-Ueno N, Ma JF (2012). Functional characterization of a silicon transporter gene implicated in silicon distribution in barley. Plant Physiol.

[3] Feng J, Shi Q, Wang X (2009). Effects of exogenous silicon on photosynthetic capacity and antioxidant enzyme activities in chloroplast of cucumber seedlings under excess manganese. Agri. Sci China.

[4] Kim YH, Khan AL, Kim DH, Lee SY, Kim KM, Waqas M, Jung HY, Shin JH, Kim JG, Lee IJ (2014). Silicon mitigates heavy metal stress by regulating P-type heavy metal ATPases, *Oryza sativa* low silicon genes, and endogenous phytohormones. BMC Plant Biol.

[5] Kim YH, Khan AL, Waqas M, Shim JK, Kim DH, Lee KY, Lee IJ (2014). Silicon application to rice root zone influenced the phytohormonal and antioxidant responsesunder salinity stress. J Plant Growth Regul.

[6] Kim YH, Khan AL, Waqas M, Shahzad R, Lee IJ (2016). Silicon-mediated mitigation of wounding stress acts by up-regulating the rice antioxidant system. Cereal Res Commun.

[7] Ma JF, Tamai K, Yamaji N, Mitani N, Konishi S, Katsuhara M, Ishiguro M, Kurata Y, Yano MA (2006). silicon transporter in rice. Nature.

[8] Hamayun M, Sohn EY, Khan SA, Shinwari ZK, Khan AL, Lee IJ. Silicon alleviates the adverse effects of salinity and drought stress on growth and endogenous plant growth hormones of soybean (*Glycine Max* L.). Pak J Bot 2010;42:1713-1722.

[9] Al-aghabary K, Zhu Z, Qinhua S (2005). Influence of silicon supply on chlorophyll content, chlorophyll fluorescence and antioxidative enzyme activities in tomato plants under salt stress. J Plant Nutr.

[10] Epstein E (1999). Silicon: Ann Rev. Plant Physiol Mol Biol.

[11] Kim YH, Khan AL, Lee IJ (2016). Silicon: a duo synergy for regulating crop growth and hormonal signaling under abiotic stress conditions. Crit Rev Biotechnol.

[12] Raven J (1983). The transport and function of silicon in plants. Biol Rev.

[13] Yamaji N, Mitatni N, Ma JFA (2008). Transporter regulating silicon distribution in rice shoots. Plant Cell.

[14] Barber SA (1995). Soil nutrient bioavailability: a mechanistic approach.

[15] Gilliham M, Dayod M, Hocking BJ, Xu B, Conn SJ, Kaiser BN, Leigh RA, Tyerman SD (2011). Calcium delivery and storage in plant leaves: exploring the link with water flow. J Exp Bot.

[16] Kudla J, Batistič O, Hashimoto K (2010). Calcium signals: the lead currency of plant information processing. Plant Cell.

[17] Zhao D, Reddy KR, Kakani VG, Reddy VR (2005). Nitrogen deficiency effects on plant growth, leaf photosynthesis, and hyperspectral reflectance properties of sorghum. Eur J Agron.

[18] Murphy CJ, Baggs EM, Morley N, Wall DP, Paterson E. Nitrogen availability alters rhizosphere processes mediating soil organic matter mineralisation. Plant Soil. 2017; 10.1007/s11104-017-3275-0.

[19] Salman D, Morteza S, Dariush Z, Nasiri A, Reza Y, Ehsan GD, Reza NNA. Application of nitrogen and silicon rates on morphological and chemical lodging related characteristics in rice (*Oryza sativa* L.) at North of Iran. J Agri Sci. 2012;4(6):12–8. 10.5539/jas.v4n6p12.

[20] Wu X, Yu Y, Baerson SR, Song Y, Liang G, Ding C, Niu J, Pan Z, Zeng R. Interactions between nitrogen and silicon in rice and their effects on resistance toward the brown planthopper *Nilaparvata lugens*. Front Plant Sci. 2017;8:28. 10.3389/fpls.2017.00028. 10.3389/fpls.2017.00028 PMC5253352 28167952

[21] Neu S, Schaller J, Dudel EG (2017). Silicon availability modifies nutrient use efficiency and content, C: N: P stoichiometry, and productivity of winter wheat (*Triticum aestivum* L.). Sci Rep.

[22] Walter A, Schurr U (2005). Dynamics of leaf and root growth: endogenous control versus environmental impact. Ann Bot.

[23] Hwang SJ, Hamayun M, Kim HY, Na CI, Kim KU, Shin DH, Kim SY, Lee IJ (2007). Effect of nitrogen and silicon nutrition on bioactive gibberellin and growth of rice under field conditions. J crop Sci. Biotech.

[24] Sivanesan, I., Jeong, B.R. Silicon promotes adventitious shoot regeneration and enhances salinity tolerance of *Ajuga multiflora* Bunge by altering activity of antioxidant enzyme. Sci World J. 2014. 10.1155/2014/521703. 10.1155/2014/521703 PMC3913090 24526904

[25] Kim YH, Khan AL, Waqas M, Lee IJ (2017). Silicon regulates antioxidant activities of crop plants under abiotic-induced oxidative stress: a review. Front Plant Sci.

[26] Cooke J, Leishman MRI (2011). Plant ecology more siliceous than we realise. Trends Plant Sci.

[27] Jang SW, Hamayun M, Sohn EY, Shin DH, Kim KU, Lee IJ (2007). Studies on the effect of silicon nutrition on plant growth, mineral contents and endogenous gibberellins of three rice cultivars. J crop Sci. Biotech.

[28] Savant NK, Snyder GH, Datnoff LE (1996). Silicon management and sustainable rice production. Adv Agron.

[29] Tripathi DK, Singh VP, Chauhan DK, Prasad SM (2017). Silicon in plants: advances and future prospects.

[30] Tripathi DK, Singh S, Singh VP, Prasad SM, Dubey NK, Chauhan DK (2017). Silicon nanoparticles more effectively alleviated UV-B stress than silicon in wheat (*Triticum aestivum*) seedlings. Plant Physiol Biochem.

[31] Ma JF (2004). Role of silicon in enhancing the resistance of plants to biotic and abiotic stresses. Soil Sci Plant Nutr.

[32] Yoshida S, Ohnishi Y, Kitagishi K (1959). Role of silicon in rice nutrition. Soil Plant Food.

[33] Lee IJ, Foster KR, Morgan PW (1998). Photoperiod control of gibberellin levels and flowering in sorghum. Plant Physiol.

[34] Kim YH, Ahn IO, Khan AL, Kamran M, Waqas M, Lee JS, Kim DH, Jang SW, Lee IJ (2014). Regulation of endogenous gibberellins and abscisic acid levels during different seed collection periods in *Panax ginseng*. Hort Environ Biotechnol.

[35] McCloud ES, Baldwin IT (1997). Herbivory and caterpillar regurgitants amplify the wound-induced increases in jasmonic acid but not nicotine in *Nicotiana sylvestris*. Planta.

[36] Enyedi AJ, Yalpani N, Silverman P, Raskin I (1992). Localization, conjugation, and function of salicylic acid in tobacco during the hypersensitive reaction to tobacco mosaic virus. Proc Natl Acad Sci U S A.

[37] Seskar M, Shulaev V, Raskin I (1998). Endogenous methyl salicylate in pathogen-inoculated tobacco plants. Plant Physiol.

[38] Choi KY, Lee YB (1999). Effect of salinity of nutrient solution on growth, translocation and accumulation of ^45^Ca in butterhead lettuce. In International Symposium on Growing Media and Hydroponics.

[39] Bradford MMA (1976). Rapid and sensitive method for the quantitation of microgram quantities of protein utilizing the principle of protein-dye binding. Anal Biochem.

[40] Blum H, Beier H, Gross HJ (1987). Improved silver staining of plant proteins, RNA and DNA in polyacrylamide gels. Electrophoresis.

[41] Carpentier SC, Witters E, Laukens K, Deckers P, Swennen R, Panis B (2005). Preparation of protein extracts from recalcitrant plant tissues: an evaluation of different methods for two-dimensional gel electrophoresis analysis. Proteomics.

[42] Feinberg AP, Vogelstein BA (1983). Technique for radiolabeling DNA restriction endonuclease fragments to high specific activity. Anal Biochem.

[43] Luyckx M, Hausman JF, Lutts S, Guerriero G. Silicon and plants: current knowledge and technological perspectives. Front Plant Sci. 2017;8: doi.org/10.3389/fpls.2017.00411. 10.3389/fpls.2017.00411 PMC5362598 28386269

[44] Song A, Xue G, Cui P, Fan F, Liu H, Yin C, Sun W, Liang Y. The role of silicon in enhancing resistance to bacterial blight of hydroponic-and soil-cultured rice. Sci Rep. 2016;6 10.1038/srep24640. 10.1038/srep24640 PMC4835757 27091552

[45] Deshmukh R, Bélanger RR (2016). Molecular evolution of auaporins and silicon influx in plants. Funct Ecol.

[46] Datnoff LE, Snyder GH, Korndörfer GH. "Silicon in agriculture." in the Elsevier, Amsterdam: Korndörfer and Lepsch. 2001. p. 133–47.

[47] Hirel B, Le Gouis J, Ney B, Gallais A (2007). The challenge of improving nitrogen use efficiency in crop plants: towards a more central role for genetic variability and quantitative genetics within integrated approaches. J Exp Bot.

[48] Hussain A, Mun BG, Imran QM, Lee SU, Adamu TA, Shahid M, Kim KM, Yun BW. Nitric oxide mediated transcriptome profiling reveals activation of multiple regulatory pathways in *Arabidopsis thaliana*. Front Plant Sci. 2016;7 10.3389/fpls.2016.00975. 10.3389/fpls.2016.00975 PMC4926318 27446194

[49] Schulten HR, Schnitzer M (1997). The chemistry of soil organic nitrogen: a review. Biol Fertil Soils.

[50] Epstein E (1972). Mineral nutrition of plants: principles and perspectives.

[51] Evans HJ, Sorger GJ (1966). Role of mineral elements with emphasis on the univalent cations. Ann rev. Plant Physiol.

[52] Mae T (1997). Physiological nitrogen efficiency in rice: nitrogen utilization, photosynthesis, and yield potential. Plant Soil.

[53] Cho YS, Jeon WT, Park CY, Park KD, Kang UG (2006). Study of nutrient uptake and physiological characteristics of rice by ^15^N and purified Si fertilization level in a transplanted pot experiment. Korean. J Crop Sci.

[54] Jawahar S, Vaiyapuri V (2010). Effect of sulphur and silicon fertilization on growth and yield of rice. Int J Curr Res.

[55] Bian R, Li L, Bao D, Zheng J, Zhang X, Zheng J, Liu X, Cheng K, Pan G (2016). Cd immobilization in a contaminated rice paddy by inorganic stabilizers of calcium hydroxide and silicon slag and by organic stabilizer of biochar. Environ Sci Pollut Res.

[56] Ma JF, Takahashi E (1989). Effect of silicic acid on phosphorus uptake by rice plant. Soil Sci Plant Nut.

[57] Kaya C, Tuna L, Higgs D (2006). Effect of silicon on plant growth and mineral nutrition of maize grown under water-stress conditions. J Plant Nut.

[58] Xi J, Qiu Y, Du L, Poovaiah BW (2012). Plant-specific trihelix transcription factor AtGT2L interacts with calcium/calmodulin and responds to cold and salt stresses. Plant Sci.

[59] Colebrook EH, Thomas SG, Phillips AL, Hedden P (2014). The role of gibberellin signalling in plant responses to abiotic stress. J Exp Biol.

[60] Reber M, Kaneta T, Kawaide H, Kamiya Y (1999). Regulaton of gibberellin biosynthesis genes during flower and fruit development of tomato. The. Plant J.

[61] Kim YH, Choi KI, Khan AL, Waqas M, Lee IJ (2016). Exogenous application of abscisic regulates endogenous gibberellins homeostasis and enhances resistance of oriental melon (*Cucumis melo* Var. L.) against low temperature. Sci Hort.

[62] Kim YH, Hwang SJ, Waqas M, Khan AL, Lee JH, Lee JD, Nguyen HT, Lee IJ (2015). Comparative analysis of endogenous hormones level in two soybean (*Glycine max* L.) lines differing in waterlogging tolerance. Front Plant Sci.

[63] Nishiuchi S, Yamauchi T, Takahashi H, Kotula L, Nakazono M (2012). Mechanisms for coping with submergence and waterlogging in rice. Rice.

[64] Shani E, Weinstain R, Zhang Y, Castillejo C, Kaiserli E, Chory J, Tsien RY, Estelle M (2013). Gibberellins accumulate in the elongating endodermal cells of Arabidopsis root. Proc Nat Acad Sci USA.

[65] Goetz S, Hellwege A, Stenzel I, Kutter C, Hauptmann V, Forner S, McCaig B, Hause G, Miersch O, Wasternack C, Hause B (2012). Role of cis-12-oxo-phytodienoic acid in tomato embryo development. Plant Physiol.

[66] He Y, Fukushige H, Hildebrand DF, Gan S (2002). Evidence supporting a role of jasmonic acid in Arabidopsis leaf senescence. Plant Physiol.

[67] Cenzano A, Vigliocco A, Kraus T, Abdala G (2003). Exogenously applied jasmonic acid induces changes in apical meristem morphology of potato stolons. Annals Bot.

[68] Yang F, Zhang Y, Huang Q, Yin G, Pennerman KK, Yu J, Liu Z, Guo A (2015). Analysis of key genes of jasmonic acid mediated signal pathway for defense against insect damages by comparative transcriptome sequencing. Sci Rep.

[69] Ahmad P, Rasool S, Gul A, Sheikh SA, Akram NA, Ashraf M, Kazi AM, Gucel S. Jasmonates: multifunctional roles in stress tolerance. Front Plant Sci. 2016;7 10.3389/fpls.2016.00813. 10.3389/fpls.2016.00813 PMC4908892 27379115

[70] Pieterse CM, Van d (2012). Does D, Zamioudis C, Leon-Reyes a, van Wees SC. Hormonal modulation of plant immunity. Ann Rev Cell Dev Biol.

[71] Currie HA, Perry CC (2007). Silica in plants: biological, biochemical and chemical studies. Ann Bot.

[72] Kim YH, Khan AL, Waqas M, Jeong HJ, Kim DH, Shin JS, Kim JG, Yeon MH, Lee IJ (2014). Regulation of jasmonic acid biosynthesis by silicon application during physical injury to *Oryza sativa* L.. J Plant Res.

[73] Gong H, Zhu X, Chen K, Wang S, Zhang C (2005). Silicon alleviates oxidative damage of wheat plants in pots under drought. Plant Sci.

[74] Kim YH, Khan AL, Hamayun M, Kang SM, Beom YJ, Lee IJ (2011). Influence of short-term silicon application on endogenous physiohormonal levels of *Oryza sativa* L. under wounding stress. Biol Trace Elem Res.

[75] Jiang CJ, Shoji K, Matsuki R, Baba A, Inagaki N, Ban H, Iwasaki T, Imamoto N, Yoneda Y, Deng XW, Yamamoto N (2001). Molecular cloning of a novel importin α homologue from rice, by which constitutive photomorphogenic 1 (COP1) nuclear localization signal (NLS)-protein is preferentially nuclear imported. J Biol Chem.

[76] Yamamoto N, Deng XW (1999). Protein nucleocytoplasmic transport and its light regulation in plants. Genes Cells.

[77] Hirota T, Tsuboi H, Iizuka-Koga M, Takahashi H, Asashima H, Yokosawa M, Kondo Y, Ohta M, Wakasa Y, Matsumoto I, Takaiwa F, Sumida T (2017). Suppression of glucose-6-phosphate-isomerase induced arthritis by oral administration of transgenic rice seeds expressing altered peptide ligands of glucose-6-phosphate-isomerase. Mod Rheumatol.

[78] Lee JH, Chang KZ, Patel V, Jeffery CJ (2001). Crystal structure of rabbit phosphoglucose isomerase complexed with its substrate D-fructose 6-phosphate. Biochemist.

[79] Das P, Seal P, Biswas AK (2016). Regulation of growth, antioxidants and sugar metabolism in rice (*Oryza sativa* L.) seedlings by NaCl and its reversal by silicon. Am. J. Plant Sci.

