# Supplementary material for: Exogenous short-term silicon application regulates macro-nutrients, endogenous phytohormones, and protein expression in Oryza sativa L
Source: BMC Plant Biol. 2018 Jan 4;18:4. doi: 10.1186/s12870-017-1216-y (PMC5755014; doi:10.1186/s12870-017-1216-y)
Supplement: Supplementary file 1 — Conditions of GC-MS-SIM and HPLC for analyzing the plant hormones. (DOCX 14 kb) [file 12870_2017_1216_MOESM1_ESM.docx]

**Table S1.** Conditions of GC-MS-SIM and HPLC for analyzing the plant hormones.

| **GC-MS condition** | | |
| --- | --- | --- |
| Equipment | Hewlett-Packard 6890, 5973N Mass Selective Detector | |
| Column | HP-1 capillary column  (30 m × 0.25 mm i.d. 0.25 µm film thickness) | |
| Carrier gas | He (40 ℃/min) | |
| Source temperature | 250℃ | |
| Oven  conditions | GA: 60℃(1 min)→15℃/min→200℃(1 min)→5℃/min→285℃(5 min)  JA:60℃(2min)→10℃/min→140℃(3min)→3℃/min→170℃→15℃/min→285℃(8 min) | |
| Injector temperature | 200℃ | |
| Ionizing voltage | 70 ev | |
| **HPLC condition** | |  |
| Equipment | Waters model 510 |  |
| Column | μ Bondapak C_18_ (3.9 mm × 300 mm) |  |
| Solvent A | 28% MeOH + 72% double distilled water (include 1% acetic acid) |  |
| Solvent B | 100% MeOH |  |
| Gradient | 100% Solvent A (0 - 5 min)→100% Solvent B (5 - 36 min)→100% Solvent B (36 - 40 min) |  |
| Flow rate | 1.5 ml/min |  |
