# Supplementary material for: Exogenous short-term silicon application regulates macro-nutrients, endogenous phytohormones, and protein expression in Oryza sativa L
Source: BMC Plant Biol. 2018 Jan 4;18:4. doi: 10.1186/s12870-017-1216-y (PMC5755014; doi:10.1186/s12870-017-1216-y)
Supplement: Supplementary file 2 — Information for GC-MS-SIM chromatogram. Bioactive GA1 showed two different ion values [2H2 GA1 (508 ion) was used as the standard and GA1 (506 ion) was used as endogenous GA1]. (DOCX 14 kb) [file 12870_2017_1216_MOESM2_ESM.docx]

**Table S2.** The primers used for real-time PCR.

| **Accession** | **Primer sequence** |
| --- | --- |
| LOC_Os06g14510 | Forward: TCCTTGGTTTGCTGAGTGTG  Reverse: AAGGACAATTGAACGCCATC |
| LOC_Os01g14950 | Forward: TTGCTGGTGATTCCCCTAAG  Reverse: AATGATGGCTGTGGTTTTCC |
| LOC_Os03g13170  (*OsUBI*) | Forward: GACGGACGCACCCTGGCTGACTAC  Reverse: TGCTGCCAATTACCATATACCACGAC |
