# Supplementary material for: Exogenous short-term silicon application regulates macro-nutrients, endogenous phytohormones, and protein expression in Oryza sativa L
Source: BMC Plant Biol. 2018 Jan 4;18:4. doi: 10.1186/s12870-017-1216-y (PMC5755014; doi:10.1186/s12870-017-1216-y)
Supplement: Supplementary file 3 — Information for GC-MS-SIM chromatogram. Me-JA retention time was 18:46 and internal standard (9,10-2H2 JA) retention time was 18:70. (DOCX 15 kb) [file 12870_2017_1216_MOESM3_ESM.docx]

**Table S3.** Influence of mineral uptake in rice plants after lone nutrient treatment or each nutrient with Si application. All plant samples were exposed to the nutrient alone or in combination with Si for 24 h and then samples were analyzed for mineral uptake.

| Treatments | T-N  (%) | P  (%) | K  (%) | Ca  (%) | Mg  (%) | Si  (ppm) |
| --- | --- | --- | --- | --- | --- | --- |
| Control | 1.32±0.20^c^ | 0.26±0.01^a^ | 1.63±0.21^a^ | 0.17±0.01^a^ | 0.11±0.01^a^ | 3,024±2.92^b^ |
| 4 mM NH_4_NO_3_ | 1.79±0.31^a^ | 0.24±0.01^a^ | 1.64±0.18^a^ | 0.17±0.01^a^ | 0.11±0.01^a^ | 3,108±18.2^b^ |
| 4 mM NH_4_NO_3_  + 1 mM Si | 1.60±0.14^b^ | 0.24±0.08^a^ | 1.63±0.14^a^ | 0.16±0.01^a^ | 0.11±0.01^a^ | 3,446±29.6^a^ |
| Treatments | T-N  (%) | P  (%) | K  (%) | Ca  (%) | Mg  (%) | Si  (ppm) |
| Control | 1.28±0.11a | 0.27±0.1a | 1.91±0.31a | 0.23±0.02b | 0.13±0.01a | 3,113±12.6b |
| 5 mM CaCl_2_ | 1.31±0.18a | 0.24±0.07a | 1.94±0.24a | 0.47±0.07a | 0.13±0.01a | 3,082±13.2b |
| 5 mM CaCl_2_  + 1 mM Si | 1.23±0.16a | 0.29±0.0.5a | 1.87±0.26a | 0.19±0.01b | 0.11±0.01a | 3,431±48.2a |

Values with ± shows the standard error of the mean of three replications. Different letters in the same column indicate significant differences at P < 0.05 according to DMRT. Statistical tests conducted for each nitrogen and calcium treatment.
