# Supplementary figures and images for: Exogenous short-term silicon application regulates macro-nutrients, endogenous phytohormones, and protein expression in Oryza sativa L
Source: BMC Plant Biol. 2018 Jan 4;18:4. doi: 10.1186/s12870-017-1216-y (PMC5755014; doi:10.1186/s12870-017-1216-y)

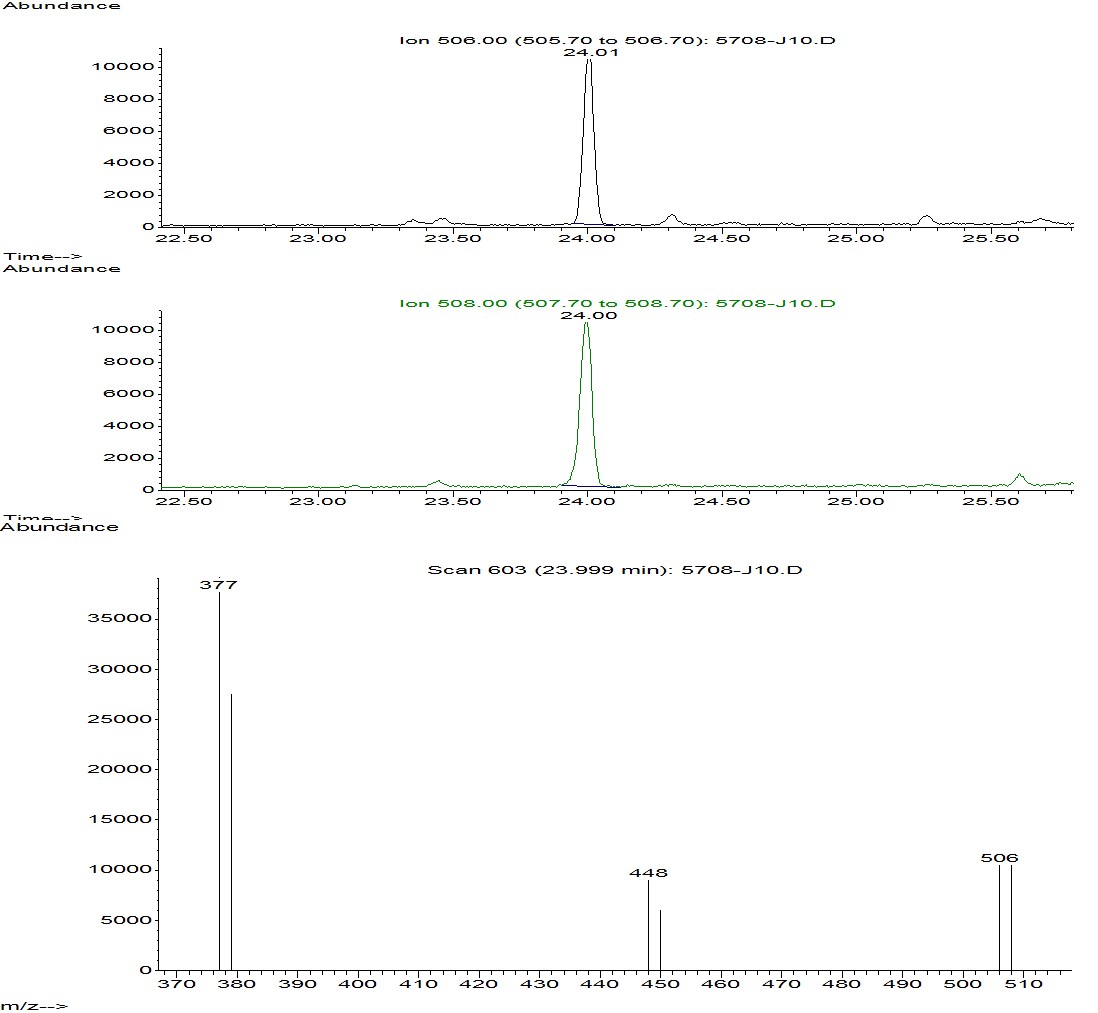

Supplement: Supplementary file 4 — The primers used for real-time PCR. (JPEG 107 kb) [file 12870_2017_1216_MOESM4_ESM.jpg]

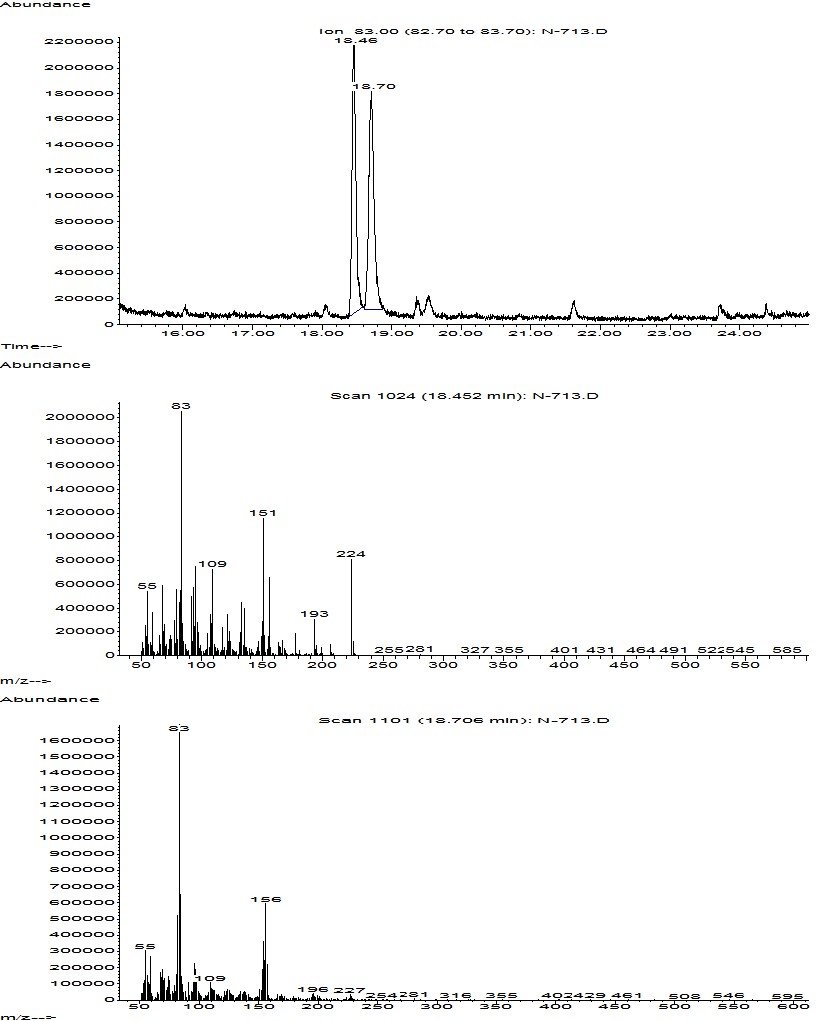

Supplement: Supplementary file 5 — Influence of mineral uptake in rice plants after lone nutrient treatment or each nutrient with Si application. All plant samples were exposed to the nutrient alone or in combination with Si for 24 h and then samples were analyzed for mineral uptake. (JPEG 107 kb) [file 12870_2017_1216_MOESM5_ESM.jpg]
